# Perisynaptic Schwann cells phagocytose nerve terminal debris in a mouse model of Guillain‐Barré syndrome

**DOI:** 10.1111/jns.12373

**Published:** 2020-04-20

**Authors:** Madeleine E. Cunningham, Gavin R. Meehan, Sophie Robinson, Denggao Yao, Rhona McGonigal, Hugh J. Willison

**Affiliations:** ^1^ Institute of Infection, Immunity and Inflammation, College of Medical, Veterinary and Life Sciences University of Glasgow Glasgow UK

**Keywords:** neuromuscular junction, Guillain‐Barré syndrome, macrophage, mouse model, perisynaptic Schwann cell

## Abstract

In mouse models of acute motor axonal neuropathy, anti‐ganglioside antibodies (AGAbs) bind to motor axons, notably the distal nerve, and activate the complement cascade. While complement activation is well studied in this model, the role of inflammatory cells is unknown. Herein we aimed to investigate the contribution of phagocytic cells including macrophages, neutrophils and perisynaptic Schwann cells (pSCs) to distal nerve pathology. To observe this, we first created a subacute injury model of sufficient duration to allow inflammatory cell recruitment. Mice were injected intraperitoneally with an anti‐GD1b monoclonal antibody that binds strongly to mouse motor nerve axons. Subsequently, mice received normal human serum as a source of complement. Dosing was titrated to allow humane survival of mice over a period of 3 days, yet still induce the characteristic neurological impairment. Behaviour and pathology were assessed in vivo using whole‐body plethysmography and post‐sacrifice by immunofluorescence and flow cytometry. ex vivo nerve‐muscle preparations were used to investigate the acute phagocytic role of pSCs following distal nerve injury. Following complement activation at distal intramuscular nerve sites in the diaphragm macrophage localisation or numbers are not altered, nor do they shift to a pro‐ or anti‐inflammatory phenotype. Similarly, neutrophils are not significantly recruited. Instead, ex vivo nerve‐muscle preparations exposed to AGAb plus complement reveal that pSCs rapidly become phagocytic and engulf axonal debris. These data suggest that pSCs, rather than inflammatory cells, are the major cellular vehicle for axonal debris clearance following distal nerve injury, in contrast to larger nerve bundles where macrophage‐mediated clearance predominates.

## 1 INTRODUCTION

In the autoimmune neuropathy, Guillain‐Barré syndrome (GBS), injury to peripheral nerves is in part caused by autoantibodies activating the complement cascade. In the acute motor axonal neuropathy (AMAN) form of GBS, the autoantibody targets are neural gangliosides, and are found with high frequency in AMAN patient sera. Evidence from patient autopsy tissue indicates complement activation occurs in GBS patients manifested by the deposition of complement products on axonal membranes and the presence of circulating terminal complement product MAC.^1^, ^2^ In addition to this, macrophage infiltration into the periaxonal space has been shown to be an early feature in spinal root analysis of fatal cases AMAN.^3^

In rabbit models of GBS, active immunisation with *Campylobacter jejuni* derived GM1‐like lipooligosaccharides or GM1 ganglioside causes delayed (35‐57 days) flaccid paralysis corresponding with an increase in serum reactivity to GM1 ganglioside. As observed in rabbit sciatic nerve, complement proteins are deposited at nodes of Ranvier, macrophages invade the periaxonal space and Wallerian‐like axon degeneration occurs.^4^, ^5^ In this model, there is evidence that the presence of macrophages may be associated rather with the recovery phase than the induction of injury.^6^

Our existing mouse models of GBS have focused on hyperacute injury (<24 hours), in which the injection of monoclonal anti‐ganglioside antibodies (AGAbs), followed by normal human serum (NHS) via intraperitoneal routes results in rapid onset respiratory failure (<4 hours following NHS as a supplementary complement source), accompanied by prominent distal motor nerve terminal and pre‐terminal nodal injury. This injury is maximally manifested in the intramuscular nerve fibres of the diaphragm muscle, due to its close proximity to the intraperitoneal injection site.^7^, ^8^ From these models, we have been able to monitor the hyperacute effects of AGAb and complement‐mediated axonal injury, demonstrating the loss of structural proteins such as axonal neurofilament (NFil), in parallel with the resultant deficits in respiratory function. We have also used these models to demonstrate the benefits of complement inhibition as a potential therapeutic pathway in GBS.

In the above mouse model of GBS, the formation of the MAC pore is essential for the acute structural and functional damage to axons. The prominence and severity of the MAC pore injury has precluded investigation of other mechanisms for complement‐mediated injury. For example, complement activation might be expected to result in the release of anaphylatoxins C3a and C5a, which are chemoattractants for phagocytic immune cells, including neutrophils and macrophages^9^ and can influence these cells to produce harmful, pro‐inflammatory cytokines.^10^ In addition, the complement products C3b iC3b and C3d are deposited on the membrane and opsonize it for phagocytosis.^9^

In the case of distal motor nerve injury, the role of perisynaptic Schwann cells (pSCs) which overlie the motor nerve terminal and constitute one third of the neuromuscular junction (NMJ) needs consideration. These cells have multiple functions including monitoring and regulation of synaptic transmission,^11^, ^12^, ^13^ formation and maintenance of the NMJ^14^, ^15^, ^16^ and a phagocytic response to axonal injury.^17^, ^18^ Following distal axonal injury induced by both laser axotomy (ex vivo) and alpha‐latrotoxin (in vivo), pSCs have also been shown to clear motor nerve terminal debris, much like their myelinating counterparts. Indeed recent studies have demonstrated that in a latrotoxin injury of the motor nerve terminal, which results in a calpain‐driven injury to the axon similar to our GBS mouse models,^19^, ^20^ pSCs not only become activated but also show evidence of debris clearance.^21^ However, longer term studies into the contribution of other phagocytic cells have not been investigated. In separate studies in which we have targeted pSCs for acute autoantibody attack, we have explored the bipartite relationship between distal axon and pSCs^22^; however, in the current AGAb‐mediated model, we describe herein, pSCs are not directly targeted and this is not considered further.

We recently investigated the acute effects of an anti‐C1q antibody on AGAb and complement‐mediated motor nerve terminal injury using our current mouse model. At this early timepoint, at which we observe acute complement‐mediated changes (6 hours), there was an insignificant effect on immune cell infiltration within the vicinity of the complement activation.^8^ This was unsurprising considering the window between complement activation and tissue analysis was only 6 hours, whereas macrophage recruitment occurs over 3 to 7 days.^23^, ^24^, ^25^

To investigate the consequences of our AGAb and complement‐mediated distal nerve injury at timepoints greater than 6 hours, we herein describe a model whereby mice survive beyond this critical window of injury severity. We investigate the contributions of neutrophils, macrophages and pSCs following injury to this specialised site, which may influence the rapid recovery seen in some cases of AMAN.^26^

## 2 METHODS

### 2.1 Mice

Male and female MacGreen mice (kindly provided by Prof. G. Graham, University of Glasgow) were used between 4 and 5 weeks of age for in vivo studies. These mice express enhanced green fluorescent protein (eGFP) driven by Csf1r gene resulting in expression in mononuclear phagocytes such as macrophages and monocytes.^27^ For ex vivo studies, B6.Cg‐Tg(Thy1‐CFP/S100B‐GFP) transgenic mice were used. These mice express cyan fluorescent protein (CFP) and green fluorescent protein (GFP) in their axons and Schwann cells, respectively.^28^, ^29^ All procedures were conducted under licence by the UK's Home Office and complied with the Animals (Scientific Procedures) Act of 1986.

### 2.2 Study design

Mice were assigned to study groups using a random team generator. All samples were analysed blinded. Sample size was estimated using an a priori power analysis with G*Power software (v 3.1.9.2), using an effect size of 4.03, an alpha error probability of .05, and a power of 0.8.

### 2.3 AGAbs and NHS

Anti‐GD1b IgG antibody (MOG‐1) was generated by immunisation of ganglioside‐deficient mice with ganglioside liposomes as described previously.^30^, ^31^ The properties of this antibody have been reported.^32^ Anti‐GD1b antibody binds strongly to neuronal tissue in mice, without any notable binding to glial cells. NHS as a source of human complement was taken from a single donor and stored in aliquots at −80°C.

### 2.4 Ex vivo injury model

Triangularis sterni (TS) muscle from B6.Cg‐Tg(Thy1‐CFP/S100B‐GFP) transgenic mice was dissected and mounted in oxygenated Ringer's solution (116 mM NaCl, 4.5 mM KCl, 23 mM NaHCO_3_, 1 mM NaH_2_PO4, 11 mM glucose, 1 mM MgCl_2_, and 2 mM CaCl_2_). Muscles were labelled with AGAb (100 mg/mL) for 1 hour at 4°C then washed and 40% NHS added for 10 minutes to cause an acute injury to motor nerve terminals. NHS was washed off and preparations were kept at room temperature in Ringer's for a further 4 hours to allow injury to develop. Preparations were fixed in 4% PFA at 4°C for 20 minutes before washing for 10 minutes each in PBS, 0.1 M glycine and PBS again. Preparations were stained with rabbit anti‐mouse synapsin (Abcam, 1/100) overnight in PBS containing 3% normal goat serum (NGS) and 0.5% Triton‐X100. In cytochalasin D (CytD)‐treated group, CytD (Sigma, 5 μg/mL) was added for 1 hour prior to the addition of antibody.

### 2.5 In vivo injury model

MacGreen mice were injected intraperitoneally with 40 mg/kg of the anti‐GD1b antibody, MOG1. This was followed 16 hours later by intraperitoneal injection of NHS (30 μL/g). The current MOG1 dose is a ~33% reduction compared with our previously published models.^8^ Control mice were either completely naïve mice, or mice which received NHS with no previous antibody injection (NHS only). Mice were either culled at 6 hours post‐NHS injection (to monitor for acute changes at the distal axon) or days 1 or 3 post‐NHS injection to assess neutrophil or macrophage recruitment respectively.

### 2.6 Plethysmography

Whole‐body plethysmography was performed (Electro‐Medical Measurement systems, Hampshire, UK) to measure changes in tidal volume as a readout of diaphragm function. Mice were habituated to the plethysmography chambers 1 to 3 days prior to the study's start. Recordings were taken before injection of antibody and at 6, 24, and 72 hours post‐injection of NHS. On each occasion, mice were left for 30 to 45 minutes to acclimatise to the chambers before recordings began.

### 2.7 Immunofluorescence staining

To assess the presence of C3c, MAC, NFil, and IgG3 at NMJs, diaphragms were snap frozen and stored at −80°C until cryosectioned. Longitudinal sections of 8 or 15 μm were collected onto Starfrost slides and stored at −20°C. For C3c and IgG3, sections were incubated in staining solution (PBS with 3% NGS) with alpha‐bungarotoxin (BTx)‐555 along with either mouse anti‐human C3c‐FITC (10 μg/mL in PBS) or anti‐mouse IgG3‐FITC (3.33 μg/mL in PBS) for 2 hours at room temperature. For MAC, tissue was incubated overnight in PBS with 3% NGS containing anti‐human C5b‐9 (2.375 μg/mL, Dako). For NFil staining, tissue was incubated in BTx for 1 hour at 4°C, rinsed, immersed in freezing ethanol at −20°C for 10 minutes before washing in PBS then incubating overnight in SMI31, an anti‐phosphorylated NFil heavy antibody (1/1500, Biolegend). Secondary antibodies were added next day for 2 hours at room temperature.

For macrophage quantitation, diaphragms were post‐fixed at 4°C in 4% PFA for 1 hour then sunk in sucrose and stored at −80°C. Sections were cut at 15 μm and stored at −20°C then stained with BTx‐555 (1/750) and mounted in Vectashield mounting media with DAPI. For neutrophils, fixed diaphragm was incubated with neutrophil marker antibody (NIMP‐R14 sc‐59 338, Santa Cruz Biotechnology) at 1:50 overnight in staining solution before secondary addition. All staining was done in either duplicate or triplicate.

### 2.8 Flow cytometry

For flow cytometry, diaphragms were digested for 1 hour in 5 mL 0.2% collagenase II (MP Biomedicals) at 37°C. Collagenase was inactivated with addition of FACS buffer (2% FCS in dPBS with 2 mM EDTA). Tissue was filtered through a 70 μm, strainer and washed (centrifugation at 400*g* at 4°C for 5 minutes). Lysis buffer was added for 1 minute to remove red blood cells and washed 2x with FACS buffer. Cell viability was determined using Fixable Viability Dye eFluor 780 (eBioscience, UK) at a dilution of 1/1000 in FACS buffer for 20 minutes at 4°C. Cells were washed and incubated with fluorophore‐conjugated antibodies at 1/200 dilution in Fc block (anti‐mouse CD16/32, BioLegend, in FACS buffer) for 20 minutes at 4°C. Antibodies used were CD45‐PE (eBioscience, clone 30‐F11), CD11b‐APC (BD pharminogen, clone M1/70), Ly6G‐PerCPCy5.5 (eBioscience, clone1A8‐Ly6g), F4/80‐eFluor 450 (eBioscience, clone BM8), NOS‐PECy7 (eBioscience, clone CXNFT), and CD206‐AF 700 (eBioscience, clone C068C2). Data were acquired using an LSRII flow cytometer (BD Biosciences) and analysed using FlowJo software (TreeStar version 7.6.5).

### 2.9 Image acquisition and quantitation

For quantification of C3c, MAC, NFil, and IgG3 presence at NMJs, images were captured using a Zeiss LSM880 microscope. The mean intensity of staining overlying the BTx area (to delineate the NMJ) was measured in each case. Analysis was performed using the Fiji distribution of ImageJ software (version 1.52b).^33^ For macrophage counts, images with BTx signal were captured using a Zeiss AxioImagerZ1 microscope and the number of macrophages (denoted by positive endogenous eGFP signal with DAPI+ve nucleus) in the entire field of view (FOV), within 50 μm of an NMJ and overlying NMJs were counted manually. For neutrophil quantification, the number of cells positive for NIMP‐R14+ve cells with DAPI+ve nuclei was counted in the same manner. In both types of quantification, images were captured and analysed blinded using the same rater for all like analysis. Fifteen images of each duplicate or triplicate were captured for each immunostained tissue section.

## 3 RESULTS

### 3.1 Low dose model of distal nerve injury provides sufficient injury while allowing mouse survival

**FIGURE 1:**
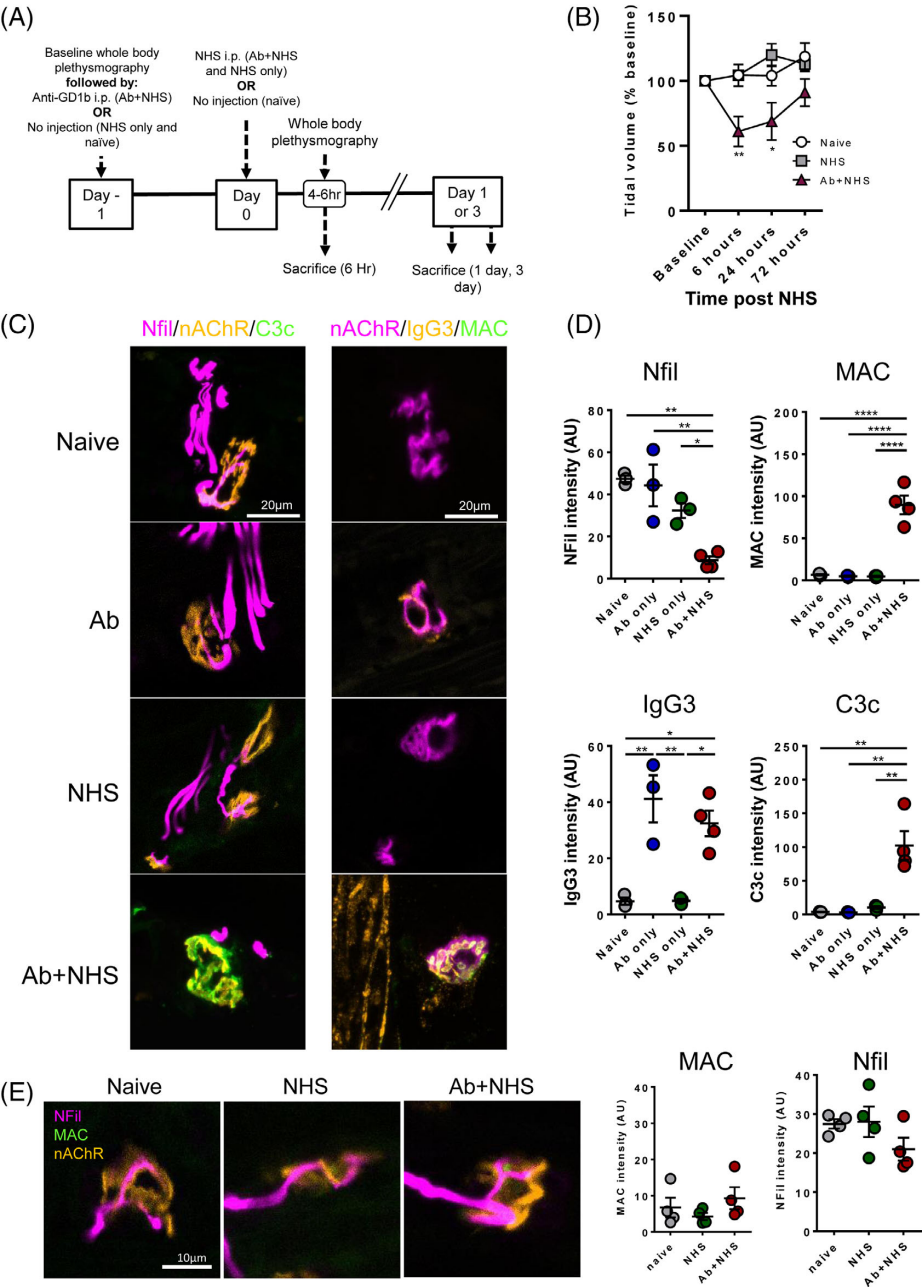
Lowered doses of anti‐ganglioside antibody (AGAb) and complement allows sufficient injury to distal nerve while allowing survival and recovery of injured mice. A, Protocol of model showing treatments given to mouse groups. B, Whole‐body plethysmography (n = 4/group) of AB + normal human serum (NHS)‐treated mice showed measurable reduction in tidal volume within 6 hours of NHS treatment vs naïve mice. After 24 hours, tidal volume was still affected but by 72 hours, it had returned to levels comparable with naive mice. NHS‐treated mice showed no difference vs naïve mice. C, Illustrative images show lack of C3c and MAC at neuromuscular junction (NMJs) of control mice while in Ab + NHS‐treated mice C3c and MAC can be seen overlying the NMJ. This corresponds with a loss of neurofilament (NFil) staining at the NMJ. D, Quantification of intensity of immunofluorescent staining for NFil, MAC, IgG3, and C3c overlying BTx signal. Ab + NHS‐treated mice showed significantly increased the presence of complement products C3c and MAC and significantly reduced NFil intensity. IgG3 was comparable between Ab only and Ab + NHS‐treated mice. E, At 3 days post‐NHS, mice from all groups had comparable levels of MAC and NFil. * = *P* < .05, ** = *P* < .01, *** = *P* < .001, **** = *P* < .0001, one‐way ANOVA with Tukey's multiple comparisons test

In our previous studies, the contribution of macrophages could not be investigated in our distal motor nerve terminal injury due to the severe nature of the respiratory injury superseding the humane endpoint for in vivo experimentation.^8^ In order to investigate recruitment of macrophages following immune‐mediate distal nerve terminal injury, the doses of AGAb and complement were reduced in comparison with our previous studies.^7^, ^8^ Otherwise, the protocol remained similar to previous studies (Figure 1A). This dose reduction allowed the survival of mice beyond the 6‐hour timepoint where robust complement activation and loss of NFil is seen but macrophage recruitment is not.^8^ Mice receiving antibody and complement still displayed the typical phenotype of impaired respiration with wasp‐like abdomen, indicating the diaphragm is paralysed. Whole‐body plethysmography also showed a reduced tidal volume in comparison to control mice, indicating an observable functional deficit (Figure 1B). Morphologically, significant amounts of complement C3c were deposited over NMJs compared with controls (Figure 1C,D). Similarly, significant MAC deposition was seen overlying the NMJ, corresponding with a significant loss in NFil heavy protein, indicating axonal injury (Figure 1C,D). Overall, this demonstrates a significant injury to the distal motor nerves was still achievable with lower doses of AGAb and complement. However, mice were able to survive past the 6‐hour post‐NHS timepoint. By the following morning (day 1), the pinched abdomen phenotype had improved and continued to recover over the subsequent days. Prior to sacrifice on day 3, their abdominal profile and tidal volume was comparable to controls. Upon sacrifice at this timepoint, NFil and MAC immunostaining was comparable between groups with infrequent NMJs from Ab + NHS‐treated mice displaying low levels of MAC (Figure 1E).

### 3.2 Neutrophils and macrophages are not recruited following AGAb and complement‐mediated distal nerve injury

**FIGURE 2:**
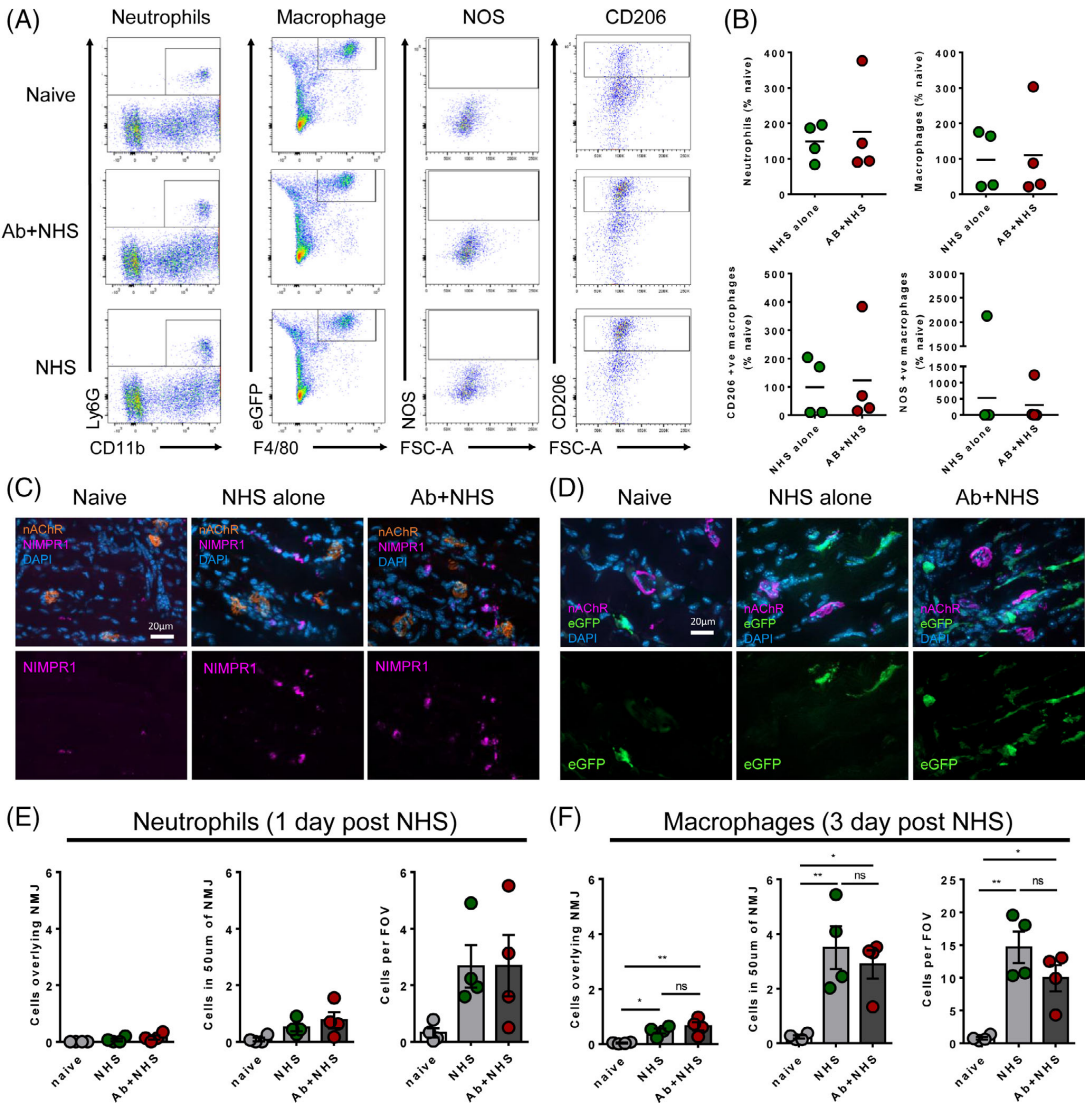
Neutrophils and macrophages do not infiltrate following anti‐ganglioside antibody (AGAb) and complement‐mediated injury to the distal nerve. A, Flow cytometry from mice survived for 3 days (n = 4 Ab + normal human serum (NHS) and NHS, n = 5 naive) showed no differences in neutrophils or macrophage numbers in the diaphragm, nor any large switch to pro‐inflammatory (NOS) or anti‐inflammatory (CD206) phenotype. B, Flow cytometry data were normalised to naïve mouse data. No differences were observed between NHS‐treated and AB + NHS‐treated mice. High variability between mice was observed. C, Illustrative examples of neutrophil immunostaining from mouse diaphragms taken 1 day after NHS. D, Illustrative examples of enhanced green fluorescent protein (eGFP) (eGFP+ve) macrophages in mouse diaphragms taken 3 days following NHS. E, Quantification of neutrophil data shows no significant changes in neutrophil numbers in close proximity to the neuromuscular junction (NMJ). F, Quantification of macrophage numbers showed increases in macrophage numbers, but this was also seen in NHS only treated animals. n = 4/group for immunofluorescence counts * = *P* < .05, ** = *P* < .01, one‐way ANOVA with Tukey's multiple comparisons test

Having established a model whereby mice could humanely survive a distal motor nerve Ab and complement‐mediated nerve injury, we examined whether immune cell recruitment occurs following such a localised injury at this specialised site. By comparing tissue taken on day 3 by flow cytometry, we found no difference in overall presence of either neutrophils or macrophages in the diaphragm muscle (Figure 2A,B). Furthermore, there were no differences in macrophages expressing either anti‐inflammatory (CD206) or pro‐inflammatory (NOS) markers, indicating no switch in phenotype of the resident tissue macrophages. As the injury site is localised to the motor nerve terminal and distal nerve, we also compared by immunofluorescence the presence of neutrophils and eGFP+ve macrophages within the proximity of the motor nerve terminals at 1 and 3 days, respectively. Specifically, we sought immune cells in the whole FOV, within 50 μm of the NMJ and overlying the NMJ. While an increase in macrophages was seen compared to naïve mice in all these categories, NHS‐treated mice also showed increased macrophage presence compared to naïve mice (Figure 2D,F). Therefore, any increases were not due to specific complement activation at site of motor nerve terminal injury, but the general presence of heterologous complement activation. Macrophages within the whole FOV were also analysed for presence of NFil staining within their cell bodies but no significant changes occurred between all three groups (data not shown). Similar trends were observed in neutrophils numbers at 1‐day timepoint (Figure 2C,E). However, these data were not statistically different, even from naïve mice.

### 3.3 pSCs phagocytose nerve terminal debris following AGAb and complement‐mediated distal nerve injury

**FIGURE 3:**
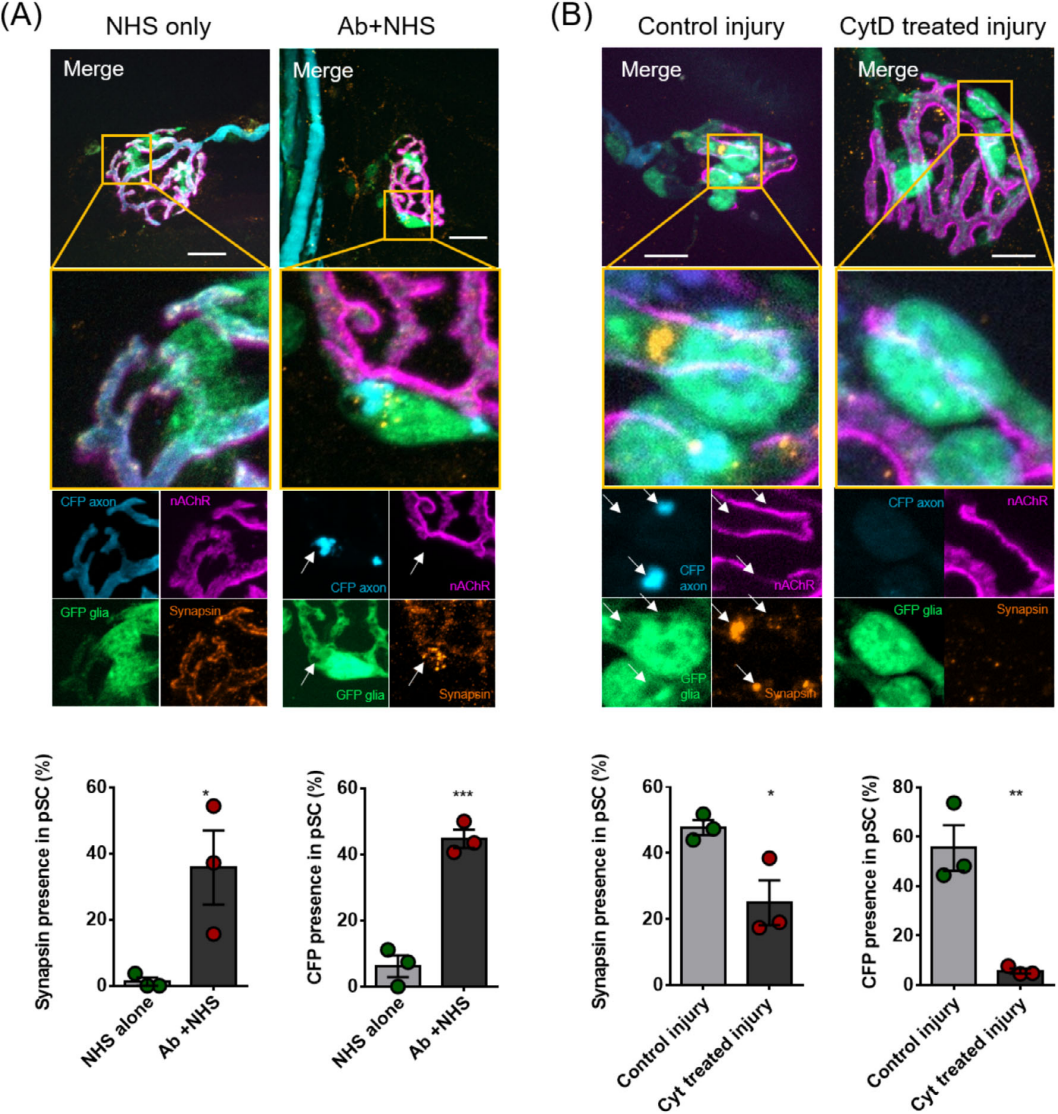
Perisynaptic Schwann cells become phagocytic and clear motor nerve terminal debris following anti‐ganglioside antibody (AGAb) and complement‐mediated injury. A, Presence of cyan fluorescent protein (CFP) and synapsin staining in control (normal human serum [NHS]) and injured (Ab and NHS) treated TS preparations, 4 hours following induction of injury (n = 3/group). Injured tissue showed globules of CFP (expressed in axonal cytoplasm) and synapsin staining within vacuoles in the cell body of the perisynaptic Schwann cell (pSC). Quantification of axonal component presence in pSC bodies after injury shows significantly higher presence of synapsin and CFP in pSCs after injury than in control preparations. B, Synapsin and CFP presence following AGAb and NHS‐mediated injury in preparations with and without cytochalasin D (CytD) treatment (n = 3/group). In CytD‐treated injury preparations, pSCs do not develop vacuoles and do not demonstrate presence of axon components. CytD‐treated preparations show significantly reduced presence of axon components CFP and synapsin in their cell bodies following AGAb and NHS‐mediated injury to the axon. * = *P* < .05, ** = *P* < .01, *** = *P* < .001, Unpaired *t* test. Scale bars = 10 μm

As we did not observe any changes in neutrophil or macrophage influx to the diaphragm relating to complement activation at the distal nerve, we investigated whether the consequences of AGAb and complement‐mediated injury may result in debris clearance by pSCs. To analyse this, we used whole mount TS, ex vivo preparations from B6.Cg‐Tg(Thy1‐CFP/S100B‐GFP) mice exposed to the MOG1 anti‐GD1b AGAb to injure the axon. Following an initial AGAb and complement‐mediated injury, components of the axon were found within pSC cell bodies after 4 hours (Figure 3A). In B6.Cg‐Tg(Thy1‐CFP/S100B‐GFP) transgenic mice, CFP which is expressed in the axonal cytoplasm and is not a substrate of the protease calpain, is found in large globules within the body of the GFP+ve pSCs. Four hours after injury, synapsin, a vesicle‐associated protein, is no longer found in a distinct pattern along the inner surface of the axon as seen in control‐treated tissue. Instead, staining for synapsin becomes more diffuse and is often found in the pSC body. These axon components are found within vacuoles in the pSCs which do not appear in the uninjured NHS‐treated tissue.

To confirm that the presence of CFP and synapsin in the pSC cell bodies resulted from phagocytosis of neuronal debris by the pSCs, ex vivo nerve‐muscle preparations were treated with CytD (which prevents actin polymerisation and therefore phagocytosis) prior to injury, then analysed for presence of components in the pSC cells bodies (Figure 3B). Treatment with CytD decreased the frequency of both CFP and synapsin presence in comparison to CytD‐untreated preparations.

## 4 DISCUSSION

In AMAN patients, macrophages are known to infiltrate into the periaxonal space in nerve roots and are thought to have pro‐ or anti‐inflammatory functions depending on the stage of disease. In our distal motor axonal injury model of GBS, we have never been able to fully investigate the late cellular consequences of complement activation.^7^, ^8^ This is due to the nature of the distal nerve injury resulting in severe respiratory paralysis that is incompatible with survival. Here, we demonstrate that by limiting the doses of AGAb and complement to an intermediate level, mice still develop a measurable injury but survive in a humane state, thereby allowing investigation of immunopathology over extended timepoints. While anti‐GD1b antibodies are most typically associated with sensory ataxic forms of GBS in humans and rabbits,^34^, ^35^ we have observed very specific and restricted binding of anti‐GD1b antibody to neuronal membranes, without any binding to glial cells, in the peripheral motor system of mice. Indeed, in our ex vivo murine preparations, it is very clearly observable that distal motor axons are severely affected with no damage to pSC cell membranes. In this sense anti‐GD1b antibody can be used to model motor axonal GBS. While there may be additional sensory deficiencies, we did not investigate these in this study. In human AMAN, axonal injury has been associated with the infiltration of macrophages at the nodes of Ranvier. In a rabbit model of AMAN, macrophages were not associated with complement‐disrupted nodes. Instead, perinodal macrophages were found in the early recovery phase, suggesting their role may be a response to injury to facilitate debris clearance, rather than an effector of pathological destruction.^6^ In our model which exhibits a very limited survival time compared with human AMAN in which supportive therapy is instituted, AGAbs primarily and initially affect the distal nerve due to absence of the blood‐nerve barrier at the motor nerve terminal. When we previously investigated macrophage presence at early timepoints in our severe, acute AMAN mouse model, no change in neutrophil or macrophage presence was seen in the vicinity of the injury site.^8^ Here, we extend these observations to show that 3 days post‐injury, no major differences in neutrophil or macrophage numbers are seen between NHS‐treated and injured mice either in the total diaphragm by flow cytometry, nor in the area directly surrounding the injury site by immunostaining. This may be due to the specific site and type of injury induced in our model, in which AGAb and complement mediate an injury that specifically targets the motor nerve terminal and distal axon. We previously observed that this distal axonal region regenerates rapidly, within a few days in mice,^36^ potentially explaining aspects of the rapid recovery seen in some cases of AMAN.^26^ This rapid regeneration precludes analysis at timepoints usually associated with immune cell infiltration, in particular macrophages whose presence peaks between 3 and 7 days post‐injury. While injury to the distal nerve does not appear therefore to require major influence from macrophages and neutrophils, a model of AMAN involving the nerve roots, another major site of injury in patients, will be useful in determining the intrinsic and extrinsic cellular consequences of complement activation by AGAbs. pSCs have been shown to phagocytose axonal debris at the synapse in other mouse models of distal nerve injury.^21^, ^37^ However, until now, longer term studies investigating additional contributions by immune cells to debris clearance after autoimmune distal nerve injury had not been investigated. Our data indicate that, while we do not rule out participation by these phagocytic cells in clearance of motor nerve terminal debris, neutrophils and macrophages do not appear to play a major role in our model of distal axon injury at the time points studied. Rather, pSCs may conduct this function in the same manner as observed in other models of distal axon injury. In our ex vivo preparations that imitate the in vivo conditions as far as is practically possible, we demonstrate that pSCs do indeed phagocytose motor nerve terminal debris following AGAb and complement‐mediated injury and this occurs within 4 hours of complement activation. This rapid phagocytic action of pSCs may explain the lack of any requirement for recruitment of phagocytic immune cells to the injury site. This implies that the distal nerve exists within a niche in which, following injury to the axon, debris clearance rapidly occurs without outside contribution, allowing for the rapid regeneration of the axon and recovery of function seen in some AMAN patients.^26^ This rapid regeneration is further exaggerated in our mouse model due to the singular and short‐lived nature of the injury, rather than an ongoing immune response as would be seen in patients whose function is maintained by intensive supportive therapies unavailable in the mouse.

This study describes a temporally extended mouse model of GBS which allows survival and recovery from distal nerve injury over a period of 3 days. This model permits observation of the longer term consequences of this injury, including recovery dynamics after therapeutic intervention and immune cell recruitment. In this model, where injury occurs primarily at the motor nerve terminal, pSCs are sufficient to clear axonal debris without any requirement for immune cell infiltration over the timepoints studied.
